# Diagnostic Accuracy of Computed Tomography Scan in Detection of Upper Gastrointestinal Tract Injuries Following Caustic Ingestion 

**Published:** 2017-03-10

**Authors:** Hooman Bahrami-Motlagh, Mohammad Hadizadeh-Neisanghalb, Hassan Peyvandi

**Affiliations:** 1 Radiology Department, Loghman Hakim Hospital, Shahid Beheshti University of Medical Sciences, School of Medicine, Tehran, Iran.; 2 General Surgery Department, Loghman Hakim Hospital, Shahid Beheshti University of Medical Sciences, Tehran, Iran.; 3 General Surgery, Hearing Disorders Research Center, Loghman Hakim Hospital, Shahid Beheshti University of Medical Sciences, Tehran, Iran.

**Keywords:** Caustics, eating, Burns, Chemical, Tomography, X-Ray Computed, Endoscopy

## Abstract

**Introduction:**

Endoscopy is an invasive procedure and finding noninvasive alternative tools in detection of probable upper gastrointestinal (GI) tract injuries following caustic ingestion is an area of interest. The present study aimed to evaluate the screening performance characteristics of thoraco-abdominal computed tomography (CT) scan in this regard.

**Methods:**

This prospective cross sectional study was conducted on patients presenting to emergency department following acute caustic ingestion. The findings of CT scan and endoscopy regarding the presence of upper GI tract damage were compared and screening performance characteristics of CT scan were calculated using MedCalc software.

**Results:**

34 patients with the mean age of 35.38±13.72 years were studied (58.8% male). The agreement rate between CT scan and endoscopy regarding the grade of esophageal and gastric injuries was moderate (K= 0.38; p = 0.001) and fair (K= 0.17; p = 0.038), respectively. The sensitivity and specificity of CT scan in detection of esophageal damage were 96.29) 79.11- 99.80) and 57.14 (20.23 - 88.19), respectively. These measures were 89.65 (71.50 - 97.28) and 40.00 (7.25 - 82.95), respectively for gastric damage. The area under the ROC curve of CT scan in detection of esophageal and gastric damages was 0.76 (95% CI: 0.52 – 1.00) and 0.64 (95% CI: 0.35 – 0.94), respectively.

**Conclusion:**

Based on the findings of the present study, CT scan could be considered as a sensitive tool in ruling out upper gastrointestinal mucosal injuries following acute caustic ingestions. However, the correlation between endoscopy and CT scan findings regarding the grading of injury is not high enough to eliminate the need for endoscopy.

## Introduction

Ingestion of caustic substances is one of the toxicology emergencies that are associated with relatively high morbidity and mortality (1). The severity of tissue damage depends on the type, concentration, volume of ingestion, and contact duration (2, 3). 

The gold standard tool in assessment of mucosal damage is esophagogastroduodenoscopy (EGD) within the first 12 hours of the incident (4, 5). 4 days after the incident, EGD is not recommended due to the risk of perforation (6, 7). Some believe that, EGD should be performed in all patients except those who have indication of emergent surgery (6, 8, 9). However, it is an invasive procedure and there are contradicting opinions about performing endoscopy in asymptomatic patients. 

Thoraco-abdominal computed tomography (CT) scan has been widely used in cases of caustic ingestion to gather more details about the surrounding tissues injury (10, 11). Lurie et al. showed that CT scan underestimates the severity of caustic-related gastrointestinal injuries compared to EGD (12). However, there is little evidence about the diagnostic accuracy of CT scan in detection of upper gastrointestinal mucosal injuries following caustic ingestion. The present study aimed to evaluate the screening performance characteristics of thoraco-abdominal CT scan in this regard.

## Methods

***Study design and setting***

This prospective cross sectional study was conducted on patients presenting to emergency department of Loghman Hakim Hospital, Tehran, Iran, in 2015, following acute caustic ingestion. All participants provided written informed consent, and the study protocol was approved by the ethics committee of Shahid Beheshti University of Medical Sciences. Researchers adhered to the Helsinki recommendations throughout the study period.

***Participant***

All adult (> 15 years old) patients, presented to the emergency department of the mentioned hospital during the study period were enrolled. This hospital is the biggest poisoning center of Tehran, Iran capital. Patients with unstable hemodynamics, third degree burns of the hypopharynx, respiratory distress, and positive history of a chronic disease or lesion in stomach or esophagus as well as those suspected to gastrointestinal perforation were excluded.

***Procedures***

Upper gastrointestinal tract endoscopy was performed by expert gastroenterologists within 24 hours of admission to hospital.

Concurrently, thoraco-abdominal CT scan with intravenous urografin 76% contrast material was carried out for all patients.

CT scan slides were reviewed by an expert radiologist who was blinded to endoscopic and clinical findings of patients. The endoscopy and CT scan grading of gastrointestinal mucosal injury were done based on appendix 1 (13, 14) .

***Data gathering***

A checklist that consisted of demographic data (sex, age), type of ingested substance (acid, alkaline), volume of ingestion, duration of hospital stay, and time from event to arriving at the hospital as well as endoscopy and CT scan grading of upper gastrointestinal injuries was used for data gathering. A trained surgery resident was responsible for collection of patients’ data. 

***Statistical analysis***

Data were analyzed using SPSS version 21. Continuous variables were presented as mean ± standard deviation and qualitative ones as frequency and percentage. The correlation between esophagus and stomach grading of injuries was calculated using Spearman rank correlation coefficient. The inter-rater agreement between CT scan and endoscopy grading was measured using calculation of Cohen's kappa coefficient (K). K=0 defined as no agreement; 0<K≤0.2 as fair; 0.2<K≤0.45 as moderate;0.45<K≤0.75 as substantial; 0.75<K≤ 1 as almost perfect; and K=1 as perfect agreement (15). 

For measuring the screening performance characteristics (sensitivity, specificity, positive and negative predictive values and likelihood ratios, and area under the receiver operating characteristic (ROC) curve) of CT scan in detection of gastrointestinal tract mucosal injuries following caustic ingestion, the endoscopy findings were considered as reference standard (5, 16). The endoscopy and CT scan findings were divided into normal or abnormal (with any grade of injury) groups. MedCalc software version 15.0 was used for calculating the screening characteristics with 95% confidence interval (CI). P < 0.05 was considered as statistically significant.

## Results

34 patients with the mean age of 35.38±13.72 years (17 – 69) were studied (58.8% male). Table 1 summarizes the baseline characteristics of studied patients. 20 (58.8%) cases were in 25 – 45 year age group and minimum and maximum volumes of caustic ingestion were 5 and 250 mL, respectively. Table 2 shows the endoscopy and CT scan grading of upper gastrointestinal tract injuries.

There was a significant correlation between esophagus and stomach grading of mucosal injuries based on endoscopy (r = 61.5; p = 0.001) and CT scan findings (r = 0.36; p = 0.036).

The agreement between CT scan and endoscopy regarding the grade of esophageal and gastric injuries was moderate (K= 0.38; p = 0.001) and fair (K= 0.17; p = 0.038), respectively. Table 3 shows the screening performance characteristics of CT scan in detection of upper gastrointestinal tract injuries following caustic ingestion. The area under the ROC curve of CT scan in detection of esophagus and stomach mucosal injuries following caustic ingestion were 0.76 (95% CI: 0.52 – 1.00) and 0.64 (95% CI: 0.35 – 0.94), respectively. 

## Discussion

Based on the findings of the present study, CT scan could be considered as a sensitive tool in screening of upper gastrointestinal mucosal injuries following caustic ingestions. In other words, CT scan could be used for ruling out mucosal injury in this setting. However, the correlation between endoscopy and CT scan findings regarding the grading of injury is not high enough to eliminate the need to perform endoscopy.

**Appendix 1:** Endoscopy and CT scan grading of upper gastrointestinal tract injuries

**Endoscopic classification based on Zargar’s grading system (14)**	
**Grade 0 **	Normal
**Grade 1**	Superficial mucosal edema and erythema
**Grade 2**	Mucosal and sub mucosal ulcerations (2A: superficial ulcerations, erosions, exudates and 2B: deep discrete or circumferential ulcerations)
**Grade 3**	Transmural ulcerations with necrosis (3A: Focal necrosis and 3B: Extensive necrosis)
**Grade 4**	Perforations
**Computed tomography grading system (13, 19, 21) **	
**Grade 1 **	No definite swelling
**Grade 2**	Edematous wall thickening without soft tissue involvement
**Grade 3**	Edematous wall thickening with soft tissue infiltration plus well-demarcated tissue interface
**Grade 4**	Edematous wall thickening with soft tissue infiltration plus blurring of tissue interface or localized fluid collection around the esophagus or descending aorta

**Table 1:** Baseline characteristics of studied patients

**variables**	**Values**
**Age (year)**	
15 - 25	7 (20.6)
25 - 35	10 (29.4)
35 - 45	10 (29.4)
45 - 55	2 (5.9)
> 55	5 (14.7)
**Sex**	
Male	20(58.8)
Female	14(41.2)
**Type of substance**	
Acid	30(88.2)
Alkaline	3(8.8)
**Volume of ingestion (mL)**	92.42 ± 89.78
**Hospital stay** **(day)**	3.88 ± 2.11
**Time to hospital (hours)**	9.69 ± 15.04

Data were presented as mean ± standard deviation or frequency and percentage

**Table 2:** Endoscopy and CT scan grading of patients’ upper gastrointestinal tract injuries

**Location**	**Grading of injuries number (%)**				
	**Normal**	**I**	**II**	**III**	**IV**
**Endoscopy**					
Esophagus	7 (20.6)	15 (44.1)	10 (29.4)	2 (5.9)	0 (0)
Stomach	5 (14.7)	5 (14.7)	21 (61.8)	3 (8.8)	0 (0)
**CT scan**					
Esophagus (proximal)	-	17 (50.0)	11 (32.4)	6 (17.6)	0 (0)
Esophagus (distal)	-	14 (41.2)	12 (35.3)	8 (23.5)	0 (0)
Stomach (fundus)	-	15 (41.64)	12 (35.3)	5 (14.7)	2 (5.9)
Stomach (body)	-	13 (38.2)	12 (35.3)	9 (26.5)	0 (0)
Stomach (antrum)		13 (38.2)	18 (52.9)	3 (8.8)	0 (0)

**Table 3:** Screening performance characteristics of CT scan in detection of upper gastrointestinal tract injuries following caustic ingestion

**Characteristics**	**Esophagus (95% CI)**	**Stomach (95% CI)**
**True positive**	26 (76.47)	26 (76.47)
**True negative**	4 (11.76)	2 (5.88)
**False positive**	3 (8.82)	3 (8.82)
**False negative**	1 (2.94)	3 (8.82)
**Sensitivity**	96.29)79.11- 99.80(	89.65 (71.50 - 97.28)
**Specificity**	57.14 (20.23 - 88.19)	40.00 (7.25 - 82.95)
**Positive Predictive Value **	89.65 (71.50- 97.28)	89.65 (71.50 - 97.10)
**Negative Predictive Value**	80.00 (29.87 - 98.94)	40.00 (7.25 - 82.95)
**Positive Likelihood Ratio**	8.66 (2.94 - 25.43)	8.66 (2.94 - 25.43)
**Negative Likelihood Ratio**	0.25 (0.03 - 1.59)	1.50 (0.41 - 5.52)

CI: confidence interval.

Currently, final decision regarding the severity of mucosal involvement of caustic ingestion is based on endoscopic findings. Endoscopy is an invasive diagnostic tools and finding noninvasive alternatives for detection of probable mucosal injuries following caustic ingestion is an area of researchers’ interest.

Bhoil’s study showed TC99m-pertechnetate has high agreement with EGD in detection of gastric injury, but this method is not affordable and available in all health centers (17).

There is little evidence regarding the accuracy of CT scan in this regard (6). Some studies have mentioned various advantages of CT scan like its availability, feasibility and ability to specify the extension of extra gastrointestinal tract involvement (10, 11, 13).

CT scan has been more valuable in the evaluation and approval of endoscopic gastric perforation (13, 18). Ryu et al. showed that CT scan has high sensitivity and specificity in predicting complications such as esophageal stricture in patients with caustic substances ingestion (19). Lurie and their colleagues assessed the role of CT scan in detecting the severity of mucosal injury due to corrosive ingestion and concluded that, decision regarding the need for surgery should not be made solely based on CT scan findings (12). In a recent review by Chirica et al. 2016, CT scan was superior to endoscopy in screening of patients in need for emergent surgery (20). 

In this study, screening performance characteristics of CT scan for detection of esophageal and gastric injuries were about the same range. Despite the high sensitivity and negative predictive value, specificity and positive predictive value of the test were not that high and therefore, this introduces CT scan as a tool for ruling out injury and not for ruling in it. The overall accuracy of the test based on area under the ROC curve is poor to moderate. These findings are in line with the results of the study by Lurie et al. (12).

In addition to being a less invasive, easy and fast method, CT scan can give important information in the field of pulmonary infiltration and surrounding thoracic soft tissue involvement. Additionally, in some situations such as upper airway inflammation, delayed visit (after 4 days and the risk of perforation), and absence of a skilled endoscopist, CT scan could be the right choice for ruling out upper gastrointestinal tract injuries. 

**Limitation:**

Low sample size was one of the limitations of this study. All CT scans were interpreted by one expert radiologist, while it was better if two radiologists reviewed the CT slides and cases of disagreement were discussed with a third radiologist.

## Conclusion:

Based on the findings of the present study, CT scan could be considered as a sensitive tool in screening upper gastrointestinal mucosal injuries following caustic ingestions. In other words, CT scan could be used for ruling out mucosal injury in this setting. However, the correlation between endoscopy and CT scan findings regarding the grading of injury is not high enough to eliminate the need to endoscopy. 
